# Supplementary material for: Poor Self-Reported Sleep is Associated with Prolonged White Matter T2 Relaxation in Psychotic Disorders
Source: bioRxiv. 2024 Jul 5:2024.07.03.601887. Preprint. [Version 1] doi: 10.1101/2024.07.03.601887 (PMC11244968; doi:10.1101/2024.07.03.601887)
Supplement: Supplement 1 [file media-1.pdf]

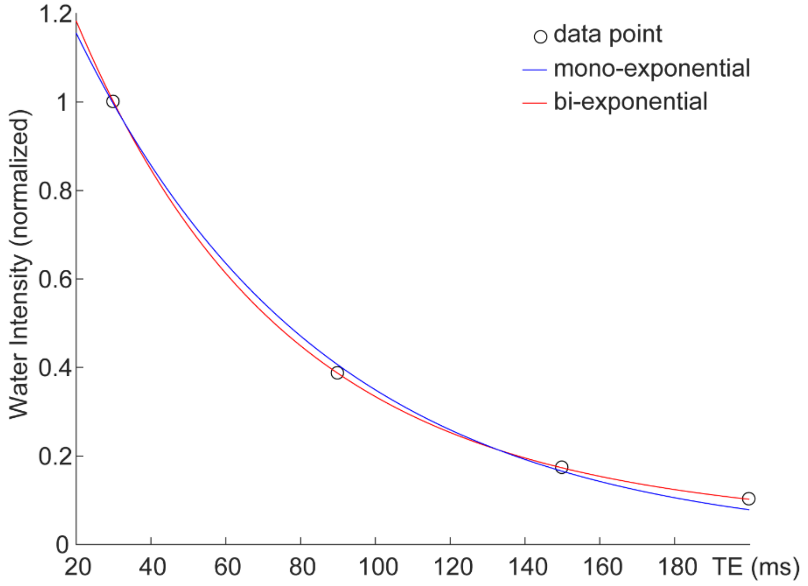

Figure S1. A representative T2 relaxation data set with mono-exponential and bi-exponential fittings

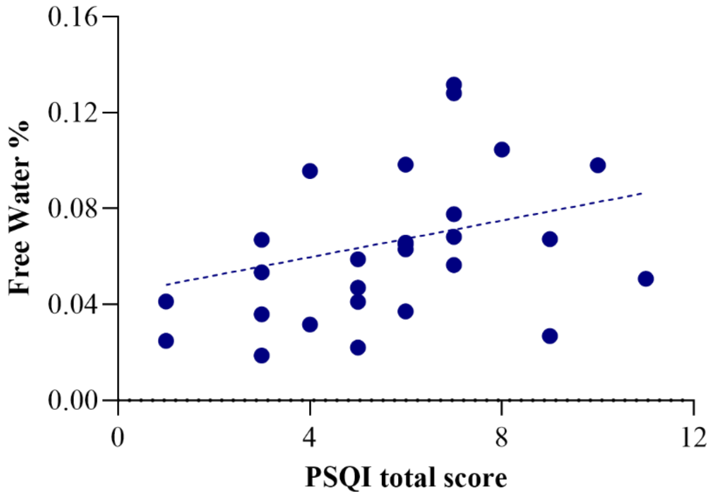

Figure S2. Correlation of PSQI total score with free water % (Spearman's rho=0.42).
